# DNAJB11 Mutation in ADPKD Patients: Clinical Characteristics in a Monocentric Cohort

**DOI:** 10.3390/genes15010003

**Published:** 2023-12-19

**Authors:** Valeria Aiello, Francesca Ciurli, Amalia Conti, Carlotta Pia Cristalli, Sarah Lerario, Francesca Montanari, Nicola Sciascia, Gisella Vischini, Benedetta Fabbrizio, Roberta Di Costanzo, Giulia Olivucci, Andrea Pietra, Antonia Lopez, Loretta Zambianchi, Gaetano La Manna, Irene Capelli

**Affiliations:** 1 Nephrology, Dialysis and Kidney Transplant Unit, IRCCS Azienda Ospedaliero-Universitaria di Bologna, 40138 Bologna, Italy; valeria.aiello6@unibo.it (V.A.); francesca.ciurli@aosp.bo.it (F.C.); gisella.vischini@aosp.bo.it (G.V.); roberta.dicostanzo@studio.unibo.it (R.D.C.); irene.capelli4@unibo.it (I.C.); 2 Department of Medical and Surgical Sciences (DIMEC), Alma Mater Studiorum University of Bologna, 40138 Bologna, Italy; sarah.lerario2@unibo.it (S.L.); giulia.olivucci@studio.unibo.it (G.O.); andrea.pietra@studio.unibo.it (A.P.); 3 Medical Genetics Unit, IRCCS Azienda Ospedaliero-Universitaria di Bologna, 40138 Bologna, Italy; amalia.conti@aosp.bo.it (A.C.); carlotta.cristalli2@unibo.it (C.P.C.); francesca.montanari2@studio.unibo.it (F.M.); 4 Pediatric and Adult CardioThoracic and Vascular, Oncohematologic and Emergency Radiology Unit, IRCCS Azienda Ospedaliero-Universitaria di Bologna, 40138 Bologna, Italy; nicola.sciascia@aosp.bo.it; 5 Pathology Unit, IRCCS Azienda Ospedaliero-Universitaria di Bologna, 40138 Bologna, Italy; benedetta.fabbrizio@aosp.bo.it; 6 Nephrology, Dialysis, Hypertension Unit, IRCCS Azienda Ospedaliero-Universitaria di Bologna, 40138 Bologna, Italy; antonia.lopez@aosp.bo.it; 7 Nephrology and Dialysis, Ospedale Nuovo Morgagni-Forlì, 47120 Forlì, Italy; loretta.zambianchi@auslromagna.it

**Keywords:** *DNAJB11*, ADPKD, atypical, mutation, genetics

## Abstract

Autosomal Dominant Polycystic Kidney Disease (ADPKD) is a late-onset cilia-related disorder, characterized by progressive cystic enlargement of the kidneys. It is genetically heterogeneous with *PKD1* and *PKD2* pathogenic variants identified in approximately 78% and 15% of families, respectively. More recently, additional ADPKD genes, such as *DNAJB11*, have been identified and included in the diagnostic routine test for renal cystic diseases. However, despite recent progress in ADPKD molecular approach, approximately ~7% of ADPKD-affected families remain genetically unresolved. We collected a cohort of 4 families from our center, harboring heterozygous variants in the *DNAJB11* gene along with clinical and imaging findings consistent with previously reported features in *DNAJB11* mutated patients. Mutations were identified as likely pathogenetic (LP) in three families and as variants of uncertain significance (VUS) in the remaining one. One patient underwent to kidney biopsy and showed a prevalence of interstitial fibrosis that could be observed in ~60% of the sample. The presence in the four families from our cohort of ADPKD characteristics together with ADTKD features, such as hyperuricemia, diabetes, and chronic interstitial fibrosis, supports the definition of *DNAJB11* phenotype as an overlap disease between these two entities, as originally suggested by the literature.

## 1. Introduction

Autosomal Dominant Polycystic Kidney Disease (ADPKD) is a late-onset cilia-related disorder, characterized by progressive cystic enlargement of the kidneys [1,2]. ADPKD can be defined as a multisystemic condition, as it presents both renal and extra-renal manifestations. The kidneys are involved with cystic bilateral enlargement, which causes other renal-related consequences such as hypertension and urological events.

ADPKD is the fourth leading cause of end-stage renal disease (ESRD) worldwide and the most common form of monogenic cystic kidney disease as it leads to kidney failure in more than 50% of patients by the age of 60. It is also the most common of all the inherited cases of chronic kidney disease (CKD) [2].

This disorder is genetically heterogeneous with *PKD1* and *PKD2* pathogenic variants identified in approximately 78% and 15% of families, respectively [3]. More recently additional ADPKD causative genes have been identified and included in diagnostic routine tests for renal cystic diseases. However, approximately ~7% of ADPKD-affected families remain genetically unresolved [4].

In 2018, Cornec-Le Gall et al. [4] first described monoallelic pathogenic variants in *DNAJB11* as a cause of cystic kidney disease, accounting for ~1.2% of ADPKD or ADPLD unresolved affected families. The clinical phenotype of patients harboring mutations in this gene was characterized by non-enlarged cystic kidneys that often evolved to kidney atrophy, while ESRD was reached later than typical genetic cases of ADPKD, from 59 to 89 years.

To date, due to the rarity of *DNAJB11* involvement in kidney diseases, limited reports are available in the literature. We present a case series of four families harboring monoallelic *DNAJB11* variants, with clinical and histological characteristics.

## 2. Materials and Methods

We retrospectively evaluated all patients referred to the Integrated Clinic of Genetic Kidney Diseases of the U.O. Nephrology, Dialysis and Transplantation at the IRCCS Azienda Ospedaliero-Universitaria of Bologna that already underwent genetic testing for clinical practice. The genetic panel performed was analyzed using Next-Generation Sequencing (NGS) technique on an Ion Torrent S5 platform. Genetic testing was carried out using a panel searching both for typical ADPKD genes (*PKD1*_NM_001009944.3 and *PKD2*_NM_000297.4) and atypical ADPKD genes: *GANAB*_NM_198335.4, *PKHD1*_NM_138694.4, *DZIP1L*_NM_173543.3, *DNAJB11*_NM_016306.5, *ALG9*_NM_001077690.1, *PRKCSH*_NM_002743.3, *SEC63*_NM_007214.5, *ALG8*_NM_024079.5, *LRP5*_NM_002335.4, *SEC61B*_NM_006808.3, *HNF1B*_NM_000458.4, *UMOD*_NM_001008389.3, *REN*_NM_000537.4, *SEC61A1*_NM_013336.4, *BICC1*_NM_001080512.3, and *IFT140*_NM_014714.4. Variants were classified in accordance with the American College of Medical Genetics and Genomics (ACMG) guidelines listing specific standard terminology: “P” for pathogenic, “LP” for likely pathogenic, “VUS” for variant of uncertain significance, “LB” for likely benign, and “B” for benign [5]. Genetic counseling was provided before and after genetic testing, according to clinical practice.

The selection criteria were the presence of a mutation in *DNAJB11*, the absence of mutations in *PKD1/PKD2*, the presence of bilateral millimetric cysts in the kidneys, and an age > 18 years. Before performing the genetic test, patients had signed an informed consent. Ethical review and approval were waived for this study because, according to the local policy, informed consent is considered sufficient for reports of an observational nature concerning a limited number of patients.

Clinical, imaging, and histological data were obtained by review of clinical records. Arterial hypertension was considered as blood pressure ≥140/90 mmHg or positive history of antihypertensive therapy, eGFR was calculated from the serum creatinine measurement using the CKD-EPI equation and expressed as mL/min/1.73 m^2^. Extra-renal involvement was evaluated by consulting clinical records, considering T2DM, renal colics, hypertension, hyperuricemia, presence of family members with ESRD, and pathological findings at the echocardiogram, as well as the presence of family members with aneurysms, or related symptoms.

The possible presence of a kidney biopsy was also assessed. When present, the routine histological staining procedures were performed on 3-μm-thick sections, including immunofluorescence (IF) studies and electron microscopy. IF was conducted on frozen sections and stained with antisera for IgG, IgA, IgM, C3, kappa, and lambda chains.

In addition, we compared data related to our cohort with the ones described by previous literature published articles.

## 3. Results

### 3.1. Study Participants and Clinical Analysis

We selected a cohort of 5 patients, belonging to 4 families and with a 5-years follow-up.

#### 3.1.1. Family 1

**Figure 1:**
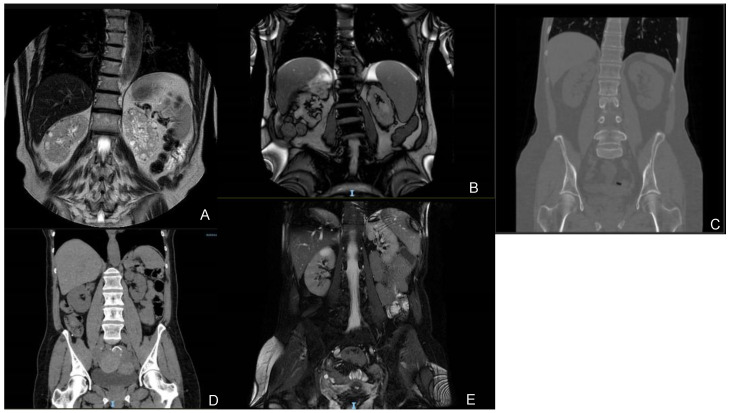
Imaging scans (**A**–**E**). (**A**) Patient 1: MRI (05/2023); (**B**) Patient 2: MRI (12/2022); (**C**) Patient 3: CT (03/2023); (**D**) Patient 4: CT (05/2023); (**E**) Patient 5: MRI (07/2020).

A 67-year-old male (Patient 1), during a follow-up for a Monoclonal Gammopathy of Undetermined Significance (MGUS) IIgG/k discovered previously, was found on September 2019 with a creatinine of 1.58 mg/dL and an eGFR of 46 mL/min/1.73 m^2^. The abdominal US and MRI showed the presence of regular-sized kidneys (both were 10 cm) with cortical millimetric cysts, while no cysts were detected in the liver (see Figure 1).

**Figure 2:**
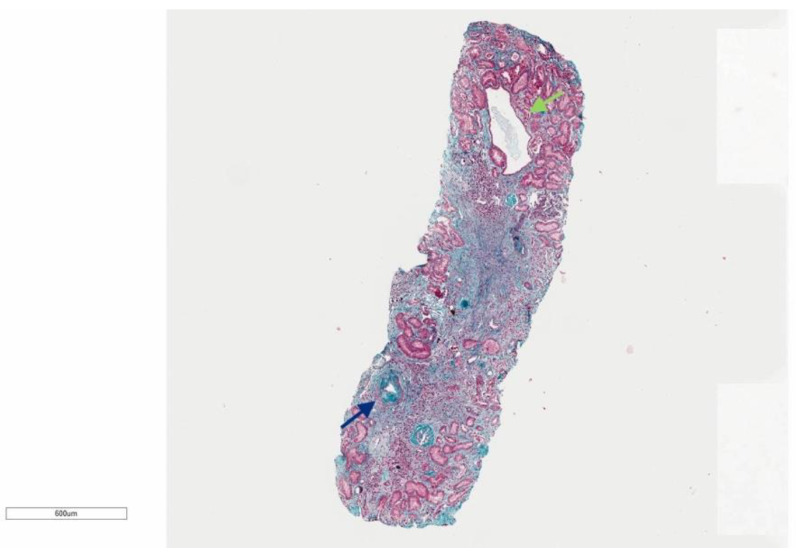
The low resolution shows severe interstitial fibrosis with slightly less tubular atrophy, several globally sclerosed glomeruli, and moderate to severe intima fibrosis of an interlobular artery (blue arrow). In addition, a mild limpho-monocytic infiltration with frequent macrophages in scared areas and one tubular cyst (green arrow) could be observed. Trichrome, magnification ×400.

A kidney biopsy was performed and analyzed with light microscopy, immunofluorescence, and electronic microscopy (see Figure 2). Multiple sections of light microscopy were prepared. The biopsy consisted of 1 piece of cortex containing up to 9 glomeruli, 7 of which were globally sclerosed, some of which are in a solidified pattern. No hypercellularity was found in the remaining glomeruli. There was a patchy disproportionate interstitial fibrosis with less tubular atrophy in around 60% of the sample and few tubular cysts. In addition, there were features consistent with moderate to severe arterionephrosclerosis, with moderate to severe thickening of tunica media and moderate intima fibrosis of interlobular arteries and arterioles. Immunofluorescence was negative for immunocomplex mediate, and immunomediate, as well as monoclonal component. Electron microscopy confirmed the features found in light microscopy and IF studies.

The subject manifested hypertension at 67 years. He presented a tendency to progressive CKD with a loss of eGFR of −4 mL/min/1.73 m^2^/year, and a serum creatinine, at the last follow-up, of 2.50 mg/dL with an eGFR of 26 mL/min/1.73 m^2^. Due to the presence of kidney cysts, he was also evaluated for extra-renal manifestations: the liver did not present any cysts, the heart was not affected by any valvulopathy, and the MRI of the brain did not show any aneurysm (only a frontal angioma was present). The patient also presented hyperuricemia. Due to the presence of an atypical cystic phenotype, genetic testing was performed to identify mutations in genes related to Autosomal Dominant Polycystic Kidney Disease (ADPKD) and other cystic nephropathies. NGS analysis showed likely pathogenic (LP) heterozygous novel variant c.716 T>G (p. Leu239Ter) in *DNAJB11*; it was absent from the population database (gnomAD), CADD 40.

**Figure 3:**
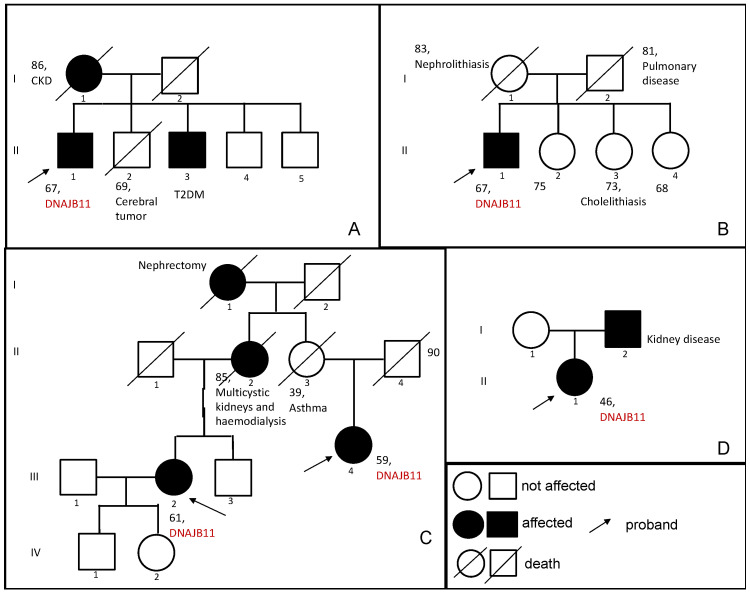
Family trees (**A**–**D**). (**A**) The first family presented two brothers (generation II, subjects 1 and 3) affected by the *DNAJB11* variant c.716 T>G (p. Leu239Ter), while their mother presented a non-diagnosed CKD. (**B**) In the second family, only the proband (generation II, subject 1) was diagnosed with the *DNAJB11* variant c.134A>G (p.Tyr45Cys), while his siblings were not available for the family segregation. (**C**) In this third family, two cousins presented the *DNAJB11* variant c.456+3_456+6del. Subject 2 of generation II also presented multicystic kidneys, although she never underwent genetic testing. Subject 3 of generation II did not show any signs or symptoms of CKD as she died at a young age. Finally, subject 1 of the generation I also presented CKD. (**D**) In the last family, subject 1 of generation II presented the *DNAJB11* variant c.499C>T (p.Arg167Trp), her father presented CKD, but he was never evaluated with genetic testing.

As for the family history (see Figure 3), his mother died at the age of 86 years, presenting a mild chronic kidney disease in her last decade of life. The proband has four brothers: one died at the age of 69 from a cerebral tumor (II-2), and another one, brother II-5, was not available for testing. The other two brothers were evaluated for the presence of the mutation: subject II-3, age 72, affected by Type 2 Diabetes Mellitus, tested positive for the familial *DNAJB11* variant and his laboratory test demonstrated a reduction of eGFR (59 mL/min/1.73 m^2^) and a slight increase in serum creatinine (1.23 mg/dL), without proteinuria (UACr < 5 mg/g). No kidney or liver cysts were detected by abdominal ultrasound. The youngest brother (II-4), age 61, did not harbor the familial *DNAJB11* variant, and his eGFR and serum creatinine were normal (87 mL/min/1.73 m^2^ and 0.94 mg/dL). Despite the lack of ultrasound renal anomalies in subject II-3, proband’s phenotype and chronic kidney disease in his family support the causative role of the variant.

#### 3.1.2. Family 2

A 67-years-old male (Patient 2) came under observation for the occurrence of nephrolithiasis. An abdominal US looking for the kidney stones highlighted the presence of regular-sized kidneys (right kidney was 11 cm, left kidney was 13 cm) with bilateral millimetric cysts. The patient later underwent an abdominal MRI (see Figure 1) that confirmed the ultrasound findings, also describing some cysts of increased dimensions (the biggest one in the right kidney with a diameter of 26 mm) while no cysts were identified in the liver. The patient also suffered from an acute myocardial infarction at 55 years, atrial fibrillation at 62 years, and hypertension. During the clinical assessment for extra-renal manifestations, an echocardiogram was performed showing the presence of a severe mitral insufficiency. He presented a tendency to progressive CKD with a loss of eGFR of −4.3 mL/min/1.73 m^2^/year. The serum creatinine at the last follow-up was 4.16 mg/dL and the eGFR was 14 mL/min/1.73 m^2^, while his uric acid was 9.3 mg/dL. NGS analysis for ADPKD and other cystic nephropathy genes identified a heterozygous likely pathogenic variant in *DNAJB11* (c.134A>G, p.Tyr45Cys), CADD 27.3.

As for family history (see Figure 3), his mother died at 83 years and suffered from nephrolithiasis throughout her life. All his three sisters are not known to be interested in any renal disease. Only one of them was reported to be affected by recurrent cholelithiasis. No sister was available for the segregation analysis.

#### 3.1.3. Family 3

A 61-years-old female (Patient 3), with a positive family history of cystic nephropathies, underwent an abdominal ultrasound during the follow-up, which found regular-sized kidneys (right kidney was 10.9, left kidney was 10.5 cm) with bilateral millimetric cysts, some of which were calcified. The liver was not involved in any cyst. A CT confirmed the absence of enlarged kidneys and liver cysts (see Figure 1). The patient presented microhematuria since the age of 47 years old and hypertension since the age of 55 years old, in addition, she suffered from recurrent cystitis and had a renal colic at 60 years old. As part of the ongoing follow-up, a mild tricuspid insufficiency was discovered by an echocardiogram in 2012, as well as a mild neurosensorial hearing loss in the left ear.

She presented a tendency to progressive CKD with a loss of eGFR of −2.3 mL/min/1.73 m^2^/year. The creatinine at the last follow-up was 1.14 mg/dL and the eGFR of 52 mL/min/1.73 m^2^. Due to the discovery of cysts in the kidneys, the patient was investigated for mutations in genes related to cystic nephropathies, and a heterozygous likely pathogenic variant in *DNAJB11* was found (c.456+3_456+6del).

As for the family history (see Figure 3), the subject’s mother presented with multicystic kidneys and died after being on hemodialysis for nine years. The maternal grandmother of the subject was probably affected by a kidney disease as well, as she underwent a nephrectomy for unknown causes. The brother underwent an ultrasound showing microlithiasis, while his renal function showed a creatinine of 0.88 mg/dL and an eGFR of 90 mL/min/1.73 m^2^. The genetic testing was negative for the family mutation.

A 59-years-old female (Patient 4) was also assessed. She is a maternal cousin of Patient 3 (see Figure 3). Her mother passed away at 39 years old due to an asthma crisis, as a result of this the presence of kidney disease could not be established. Patient 4 underwent an abdominal ultrasound that showed the presence of cysts in the kidneys and the liver: the kidneys had a normal size (right one of 10.3 cm and left one of 9.4 cm) and presented bilateral millimetric cysts, some of which were calcified. A CT was also performed confirming the ultrasound discoveries (see Figure 3).

She has suffered from hypertension since she was 50 years old. Her serum creatinine at the time of the evaluation was 1.27 mg/dL and her eGFR was 46 mL/min/1.73 m^2^. She was tested for the *DNAJB11* variant found in her cousin (c.456+3_456+6del), with a positive result.

#### 3.1.4. Family 4

A 46-years-old female (Patient 5) came under observation because she developed hypokalaemia and long QT, probably as a result of the anti-depressant drugs that she was on, and was, consequently, hospitalized at our center. Before being admitted to the hospital, indeed, she stayed in a psychiatric facility for a reactive anxiety depressive syndrome for which she was being treated. During her hospitalization her kidneys were assessed with a CT and an MRI, both showing the presence of regular-sized kidneys (both were 10 cm) with bilateral millimetric cysts (the biggest one of 33 mm) (see Figure 3). The liver presented just one cyst in the III segment with a diameter of 4 mm. The serum creatinine was 1.54 mg/dL and the eGFR was 40 mL/min/1.73 m^2^, and she also presented hyperuricemia. Genetic testing for cystic nephropathies was performed and found a heterozygous c.499C>T (p.Arg167Trp) variant, classified as a variant of unknown significance; CADD 31. Her family history (see Figure 1) showed the presence of renal disease in the father, who was not available for an assessment. The patient did not carry on a follow-up, so longitudinal data are not available.

A summary of the main clinical, imaging, and laboratory characteristics of all patients can be found in Table 1. A specific section was dedicated to the extra-renal involvement, highlighting how *DNAJB11*-ADPKD can be described as a multisystemic syndrome.

## 4. Discussion

ADPKD is an inherited multisystemic condition characterized by the presence of cysts in the kidneys, often in the liver, and many other sites. It is associated with an increased kidney volume, due to the cysts’ growth. The Pei’s criteria, [6] based on the number of cysts for each kidney and the patient’s age, allow the clinical diagnosis. The Mayo Clinic Imaging Classification created two classes according to the imaging criteria [7] differentiating typical and atypical forms. Class 1 represents a typical imaging pattern, defined by bilateral and diffuse cyst distribution causing nephromegaly. By contrast, class 2 is characterized by a unilateral, asymmetric, segmental, lopsided, or bilateral cystic distribution with unilateral or bilateral kidney atrophy. Typical and atypical imaging are directly related to the genotype [8]. Indeed, *PKD1* and *PKD2* are the two more frequent mutated genes and are linked to a typical aspect at the CT and MRI scans. On the other hand, genes described as “atypical”, such as *GANAB*, *PKHD1*, *DZIP1L, ALG8,* and *SEC61A1*, are associated with Class 2 according to the Mayo Classification.

Pathogenic variants in the *DNAJB11* gene have been recently discovered as a cause of cystic kidney disease. The clinical phenotype related to this condition has been described in previous articles, but, being this a rare mutation, there is still a need for case reports that permit to better description of clinical aspects in patients harboring the mutation. *DNAJB11* (located on chromosome 3q27.3) encodes a 358-amino acid soluble glycoprotein called ERdj3, which functions as a binding immunoglobulin protein (BiP) co-chaperone, playing a central role in the maintenance of the endoplasmic reticulum (ER) protein homeostasis [9]. BiP (also known as GRP78) is, indeed, a heat shock protein chaperone required for the proper folding and assembly of proteins in the ER. It targets misfolded proteins to ER degradation and acts as a master regulator of the unfolded protein response, an adaptive cellular response to ER stress [10]. *DNAJB11*-protein interacts with BiP through a highly conserved J domain, with a characteristic His-Pro-Asp (HPD) motif [10]. The altered mechanism in protein folding is the cause of a loss of maturation and appropriate localization of polycystin-1, the protein encoded by PKD1, resulting in renal and liver cystogenesis [3,4,11]. Polycystin-1 is particularly susceptible to dosage reduction of proteins involved in maturation and trafficking in the ER, leading to a loss of function, which was described as the main mechanism of initiation and enlargement of kidney and liver cysts [3,11,12,13].

The literature, as for the phenotype of patients with *DNAJB11* mutations, described non-enlarged cystic kidneys, together with ESRD reached later in life than typical ADPKD. In addition to these features, interstitial fibrosis, evident in non-cystic parenchyma, as well as recurring episodes of gout and hyperuricemia could also be found in these patients. The liver, unlike in typical ADPKD, has been reported to be less involved [4]. These elements indicate that *DNAJB11* is the responsible mutated gene for an atypical dominant form of ADPKD [4].

Four variants were detected in our cohort, three of which were defined as likely pathogenic and the other one as a variant of uncertain significance. Patients did not show mutations in any gene analyzed in the cystic nephropathies’ panel, apart from *DNAJB11*. To better classify the role of the variant described in Family 1, we carried out a segregation familial analysis: the proband’s brother, who tested positive for the familial variant, did not show any cyst at the ultrasound even if his serum creatinine was increased. However, a second level of imaging has not been performed yet, and this could be decisive in the discovery of millimetric kidney cysts, not detectable at the ultrasound. The kidney biopsy performed on Patient 1 was another important tool to better define the disease. It showed a disproportionate presence between interstitial fibrosis and tubular atrophy, with a prevalence of interstitial fibrosis that could be observed in ~60% of the sample. Moreover, the presence of macrophages was another important discovery, as their presence can be related to the continuous production of fibrotic material with a never-ending mechanism. A kidney biopsy, performed in a previous study on a patient with a mutation in *DNAJB11*, similarly showed the presence of tubular cysts and moderate arteriosclerosis, but much less interstitial fibrosis [14]. Therefore, the result from the biopsy of Patient 1 was unexpected since he manifested hypertension at a relatively old (67 years) age. The histological aspect should thus be further examined in other patients carrying the mutation. The family presenting the VUS (Family 4) could not be further assessed, as the subjects were not available for the follow-up. The patient was evaluated just on the occasion of her hospitalization and did not come back for further clinical assessments. This is the main reason for which the segregation was not performed on her father and her family history was not better evaluated. This family was included in the description, and the VUS was described, as the clinical and imaging aspects of Patient 5 are consistent with the other patients. Indeed, kidneys were observed as having a normal size and interest by bilateral millimetric cysts, and the serum creatinine was elevated, as in all the other cases, strengthening the idea of a possible pathogenic role of this VUS. Further descriptions of affected families and functional studies in the future may help to better elucidate the pathogenetic role of this specific variant, as a certainty of pathogenicity could not be reached after our analysis, given all the limitations previously described.

We compared the characteristics of patients from our center with those of cohorts previously described in the literature. The first study (Cornec-Le Gall et al. [4]) analyzed 722 subjects from 694 genetically unresolved ADPKD (502) and ADPLD (192) families, identifying 23 patients belonging to 7 families that harbored a mutation in *DNAJB11*. A second study (Huynh et al. [10]) described 20 new pedigrees (54 affected individuals), while a third (Pisani et al. [15]) analyzed the differences between 23 families (42 patients) affected by mutations in *PKD1*/*PKD2* and 6 families (27 patients) affected by mutations in *DNAJB11*. A summary of the main clinical, imaging, and laboratory characteristics of these three cohorts, together with the results from the Bologna cohort, is provided in Table 2.

Regarding the kidney imaging, the whole Bologna cohort presented normal-sized kidneys, with a mean diameter of 10.7 ± 1.2 cm, and bilateral small cysts. These data are consistent with the previously published cohorts (see Table 2), where bilateral small cysts in normal-sized kidneys were present in 78.3%, 83.3%, and 81.5%, respectively. The liver, on the other hand, was observed interested by cysts in fewer cases (~40%) in every cohort, strengthening the hypothesis of minor liver involvement in atypical ADPKD forms caused by this specific mutation.

Hypertension showed a higher prevalence in the Bologna cohort (80.0% of cases), against 47.8% (Cornec-Le Gall et al. [4]), 57.4% (Huynh et al. [10]), and 59.3% (Pisani et al. [15]) in the other cohorts. In all cases the age at occurrence was significantly higher than 35 years old, the age identified as a cut-off to classify rapid progressor patients in ADPKD [16].

Subjects from the Bologna cohort presented an average age of 60 ± 8.6 years and none of them had reached ESRD at the time of our evaluation, while patients from the literature showed an eGFR < 15 mL/min/1.73 m^2^ during the 8th decade, several years later than typical ADPKD patients. In the patients from Bologna, a mean value of eGFR of 35.6 ± 15.5 mL/min/1.73 m^2^ was observed with a mean loss of eGFR per year of −3.5 ± 1.1 mL/min/1.73 m^2^. Three out of the five probands that were evaluated in the present study had a follow-up > 4 years, showing a typical ADPKD rapid progressor behavior with an eGFR loss > 3 mL/min/1.73 m^2^/year, according to the ErkNet Position Statement of 2022 [17]. However, in consideration of the normal TKV and the absence of liver cysts, the Bologna cohort could be considered analogous to the atypical form of ADPKD.

Finally, we compared the presence of associated conditions among patients belonging to the different cohorts. Nephrolithiasis, hyperuricemia, and cardiovascular defects were the features most present in all cohorts.

A pathogenic variant in *DNAJB11* was observed in the literature to be associated with the retention of uromodulin in the cytoplasm caused by an altered mechanism in folding and cellular trafficking of proteins, also related to a mutation in *UMOD* [4,14]. This mechanism could be associated with the recurrence of hyperuricemia and Type 2 Diabetes Mellitus in these patients. Pisani et al. [15], in addition, showed how this feature could be found only in patients with the *DNAJB11* mutation, while not in patients with ADPKD. An in vivo study [18], on the contrary, demonstrated the absence of change in uromodulin processing in Dnajb11 knock-out mice, while showing an impaired cleavage of polycystin-1 dosage-dependent cystogenesis. Another in vitro study [19], consistent with the latter, demonstrated that the excretion, maturation, and trafficking of uromodulin are unchanged in patients with *DNAJB11* mutations. Further functional studies are needed to better define the pathophysiology behind the disease caused by a mutation in *DNAJB11*.

In conclusion, *DNAJB11* mutation, according to the findings in patients from the literature and our cohort, is related to a specific cystic disease phenotype. Moreover, the association between hyperuricemia, diabetes, and the eGFR time-dependent decrease, together with the presence of chronic interstitial fibrosis at the biopsy and of small renal cysts, indicates that this condition is characterized by partial phenotypic overlap between ADPKD and autosomal dominant tubulointerstitial diseases (ADTKD) [4]. Furthermore, the prevalence of characteristics defining an atypical disease must be highlighted. The small number of patients analyzed by the literature and implemented by our cohort, and the follow-up data that were not complete for every patient suggest that a more complete and larger assessment is necessary to draw a more definitive conclusion.

## Figures and Tables

**Table 1:** Summary of characteristics of the 5 patients described in the cohort from Bologna. In the DNAJB11 mutation column variants are expressed with their characteristics: HGVS classification; ACMG nomenclature; population frequency. N/A means Not Assessed.

	DNAJB11 Mutation	Kidney Imaging (Diameter in cm of Right/Left Kidney)	Liver Imaging	Hypertension (age)	Renal Function at Diagnosis	Renal Function at Last Follow-Up	Δ eGFR per year	Other Conditions
Patient 1 (67)	NM_016306.5:c.716T>G p.(Leu239Ter) 4 /	Regular-sized kidneys (10/10) Bilateral cortical millimetric cysts	No cysts	Yes (67)	Creat. 1.58 mg/dL eGFR 46 mL/min/1.73 m^2^ CKD IIIa	Creat. 2.50 mg/dL eGFR 26 mL/min/1.73 m^2^ CKD IV	−4	MGUS, mild bilateral perceptive hearing loss, hyperuricemia
Patient 2 (67)	NM_016306.5:c.134A>G p.(Tyr45Cys) 4 gnomAD Exomes: Version: 2.1.1ƒ = 0.00000797; gnomAD GenomesVersion: 2.1.1ƒ = 0.0000319	Regular-sized kidneys (11/13) Bilateral millimetric cysts and some bigger cysts	No cysts	Yes	Creat. 2.45 mg/dL eGFR 27 mL/min/1.73 m^2^ CKD IV	Creat. 4.16 mg/dL eGFR 14 mL/min/1.73 m^2^ CKD V	−4.3	Nephrolithiasis, AMI, AF, severe mitral insufficiency
Patient 3 (61)	NM_016306.5:c.456+3_456+6del (IVS4) 4 /	Regular-sized kidneys (10.9/10.5) Bilateral millimetric cysts	No cysts	Yes (55)	Creat. 1.04 mg/dL eGFR 59 mL/min/1.73 m^2^ CKD IIIa	Creat. 1.14 mg/dL eGFR 52 mL/min/1.73 m^2^ CKD IIIa	−2.3	Microhaematuria, recurrent cystitis, nephrolithiasis, mild tricuspid insufficiency, mild neurosensorial hearing loss in the left ear
Patient 4 (59)	NM_016306.5:c.456+3_456+6del (IVS4) 4 /	Regular-sized kidneys (10.3/9.4) Bilateral millimetric cysts	Multiple cysts	Yes (50)	Creat. 1.27 mg/dL eGFR 46 mL/min/1.73 m^2^ CKD IIIa	Creat. 1.27 mg/dL eGFR 46 mL/min/1.73 m^2^ CKD IIIa	N/A	/
Patient 5 (46)	NM_016306.5:c.499C>T p.(Arg167Trp) 3 gnomAD ExomesVersion: 2.1.1ƒ = 0.00000398; gnomAD GenomesVersion: 2.1.1 /	Regular-sized kidneys (10/10) Bilateral millimetric cysts	One cyst in the III segment (4 mm)	No	Creat. 1.54 mg/dL eGFR 40 mL/min/1.73 m^2^ CKD IIIa	Creat. 1.54 mg/dL eGFR 40 mL/min/1.73 m^2^ CKD IIIa	N/A	Hyperuricemia, reactive anxiety depressive syndrome

**Table 2:** Summary of characteristics of patients described in literature and patients described in the cohort from Bologna.

	Kidney Imaging		Liver Imaging	Hypertension	Laboratory		Other Conditions
	Presence of cysts	Mean diameter			CKD	ESRD	
Cornec- Le Gall et al. [4]	MBSC: 78.3% Unilateral cysts: 8.7% No cysts: 4.3% N/A: 8.7%	10.3 ± 1.7 cm N/A: 13.0%	Cysts: 39.1% No cysts: 47.8% N/A: 13.1%	47.8% 56.3 ± 8.4 years	69.6% 53.4 ± 17.3 years eGFR: 75.1 ± 25.3 mL/min/1.73 m^2^	30.4% 77 ± 10.8 years	Nephrolithiasis: 8.7% T2DM: 8.7% Hyperuricemia: 13.0%
Huynh et al. [10]	MBSC: 83.3% Polycystic kidneys: 5.6% No cysts: 1.8% N/A: 9.3%	12.2 ± 2.2 cm N/A: 35.2%	Cysts: 35.2% No cysts: 40.7% N/A: 24.1%	57.4% 55.1 ± 12.1 years	55.6% 58.1 ± 11.1 years eGFR: 52.7 ± 31.6 mL/min/1.73 m^2^	44.4% 71.6 ± 6.8 years	T2DM: 11.1% Hyperuricemia: 5.6% ICA: 3.7% Thoracic aortic aneurysms: 3.7%
Pisani et al. [15]	Cysts: 81.5% No cysts: 18.5%	10.3 ± 1.6 cm N/A: 18.5%	Cysts: 40.7% No cysts: 44.5% N/A: 14.8%	59.3%	N/A	44.4% 71.1 ± 4.5 years	Nephrolithiasis: 59.3% T2DM: 18.5% Cardiac valvular defects: 3.7%
Bologna cohort	MBSC: 100.0% No cysts: 0.0% N/A: 0.0%	10.7 ± 1.2 cm N/A: 0.0%	Cysts: 40.0% No cysts: 60.0% N/A: 0.0%	80.0% 57.3 ± 8.7 years	100.0% 60.0 ± 8.6 years eGFR: 35.6 ± 15.5 mL/min/1.73 m^2^	0.0 %	Nephrolithiasis: 40.0% Hyperuricemia: 40.0% Cardiac valvular defects: 40.0%

## Data Availability

All data are contained within the article.
